# General Public Preferences for Allocating Scarce Medical Resources During COVID-19

**DOI:** 10.3389/fpubh.2020.587423

**Published:** 2020-12-11

**Authors:** Samir Huseynov, Marco A. Palma, Rodolfo M. Nayga

**Affiliations:** ^1^Texas A&M University, College Station, TX, United States; ^2^University of Arkansas, Fayetteville, AR, United States

**Keywords:** scarce, ventilators, triage, principles, ethics

## Abstract

COVID-19 has overwhelmed healthcare systems across the globe with an unprecedented surge in the demand for hospitalizations. Consequently, many hospitals are facing precarious conditions due to limited capacity, especially in the provision of ventilators. The governing ethical principles of medical practice delineated in (1) favor prioritizing younger patients, largely because of their relatively higher expected life years. We conduct a survey of the general public in the United States to elicit their preferences for the allocation of a limited number of ventilators. The results show that the general public views align with the established ethical principles, which favor younger patients.

**JEL Classification**: C91.

The catastrophic consequences of COVID-19 to human health have been felt on a global scale. The virus has already impacted the health of millions and claimed the lives of several hundred thousand people across 215 countries (2). Even in developed nations, the pandemic has overwhelmed healthcare systems with an unprecedented increase in the demand for hospitalizations. Disruptions in the global supply chain for healthcare equipment, which plays a vital role in the replenishment of health-provision, have consequently left many hospitals in precarious conditions due to limited capacity and urgent needs for medical resources (1, 3, 4). The most severe shortages have been experienced in the provision of ventilators, which are essential medical equipment for treating coronavirus patients (5). This situation is exacerbated in developing countries where the public health systems tend to have more limited capacity constraints.^1^ Many countries report that medical personnel have been forced to make difficult rationing decisions regarding which patients will be assigned to ventilators or other life-saving equipment (1, 6, 7). Hospitals operating beyond capacity and severe shortages of essential resources raise the importance of the ethical considerations in determining the underlying principles and values for the fair allocation of medical treatment during COVID-19. Historically, these ethical decisions have mainly taken place during extraordinary times of warfare or heavy armed conflicts (8). The derived lessons from the COVID-19 experience can provide invaluable insights in the event of future pandemics, natural disasters or other phenomena that creates excessive burdens in the healthcare system.

## 1. Principles for Fair Allocation of Scarce Medical Resources

There is a growing interdisciplinary literature on the investigation of the main governing principles for limited medical resource allocations during pandemics (9–11). Especially, the vast medical literature identifies four main governing principles: (1) *Treating patients equally*, (2) *Prioritizing the worst-off*, (3) *Maximizing social benefits*, and (4) *Maximizing individual benefits* (1). Since the fatality rate of the coronavirus greatly varies across age groups and comorbidities, treating patients equally can only be applied among patients who have similar prognosis (1, 12). The principle of “Prioritizing the worst-off” or the allocation of limited medical resources to the sickest patients can be operationalized when it maximizes the expected post-treatment life-years (1, 13). In the context of COVID-19, this concept favors younger patients when it helps to contain the virus (assuming that younger patients are more mobile and can widely spread the virus), or the sickest patients if it maximizes survival years after the treatment. The “Maximizing social benefits” principle favors patients who provide direct benefits to communities, such as healthcare workers or research participants.^2^ However, determining which patient can provide the highest benefit to society can be extremely difficult, particularly during the course of urgent clinical decisions (1). Nevertheless, having more expected life years also increases the expected social benefits from the treated patients and favors younger patients. In contrast, older patients should be prioritized in vaccination, as the survival rate of younger patients is higher for the same waiting period (1). The principle of “Maximizing individual benefits” requires using scarce resources either for increasing the number of lives saved or for increasing post-treatment life-years, both of which generally favor younger patients (1, 14, 15).

Based on the four mentioned principles, Emanuel et al. (1) recommend that if patients have similar severity of COVID-19 symptoms, life-saving equipment and resources should be allocated to younger patients who are estimated to have the same prognosis as older patients. This resource allocation approach will maximize the benefit from post-treatment life-years (1). However, relying on on-site prognosis estimations can be problematic. Previous work has shown that physicians consistently demonstrate inaccurate prognosis estimations, which makes incorporating their judgments of survival probabilities into triage decisions very questionable (16). Therefore, in this study we simplify our context to exclusively focus on severity of observed symptoms as the main decision criteria in the allocation of scarce medical resources. Emanuel et al. (1) also highlight the importance of scrutinizing these values with the affected parties, including the general public, to ensure consensus for the fair allocation of scarce medical resources. Information about the general public's preferences for allocation of scarce medical resources such as ventilators is important and can help guide public health experts and policy makers. Our study answers to this important call and investigates public preferences over the fair distribution of limited medical resources.

## 2. Survey Details

Our study answers (1)'s call by using a survey to measure the U.S. general public views on the fair allocation of ventilators among patients who have similar morbidities and experience similar severity of COVID-19 symptoms. We employed the consequentiality method to increase the truthfulness of survey responses (17). Specifically, we partnered with public health organizations and informed survey participants that their feedback would be communicated to relevant Government offices and would affect their decisions. We conducted an online survey with 586 U.S. participants using the MTurk platform on April 6, 2020, when the COVID-19 pandemic was spreading rapidly across the United States. We restricted our online survey target audience to U.S. residents who were at least 18 years of age. We inquired responses from 600 MTurk users, and after the elimination of 14 incomplete survey entries, we ended up with the data of 586 respondents. Our sample constitutes a wide range of socio-demographic characteristics (see Table A1). The final sample has a larger proportion of males (60%) and the average age of survey respondents is 37. We controlled for gender in our regression analyses to disentangle the noise stemmed from the overrepresentation of males in our sample.

The participants were presented with a hypothetical scenario, in which 1,000 COVID-19 patients, with a similar level of severity of observed symptoms, were seeking treatment in a hospital. Since the current state of the medical ethics literature overwhelmingly prioritizes patients based on age considerations, our main focus is the age of the patients. Each respondent was asked to allocate 100 available ventilators among patients with similar symptoms that differed in age across 10 age categories, ranging from “0 to 10” years old to “90 or older” groups (see Figure A1). We partnered with public health officials and emergency disaster responding agencies and informed participants that their aggregate responses would be shared with Government officials.^3^ Providing respondents with an opportunity to voice their opinions to policy-makers over the utilization of limited medical resources enabled us to incentivize participants to respond truthfully regarding their opinion on the fair allocation of scarce medical resources during COVID-19.

## 3. Main Findings

**Figure 1:**
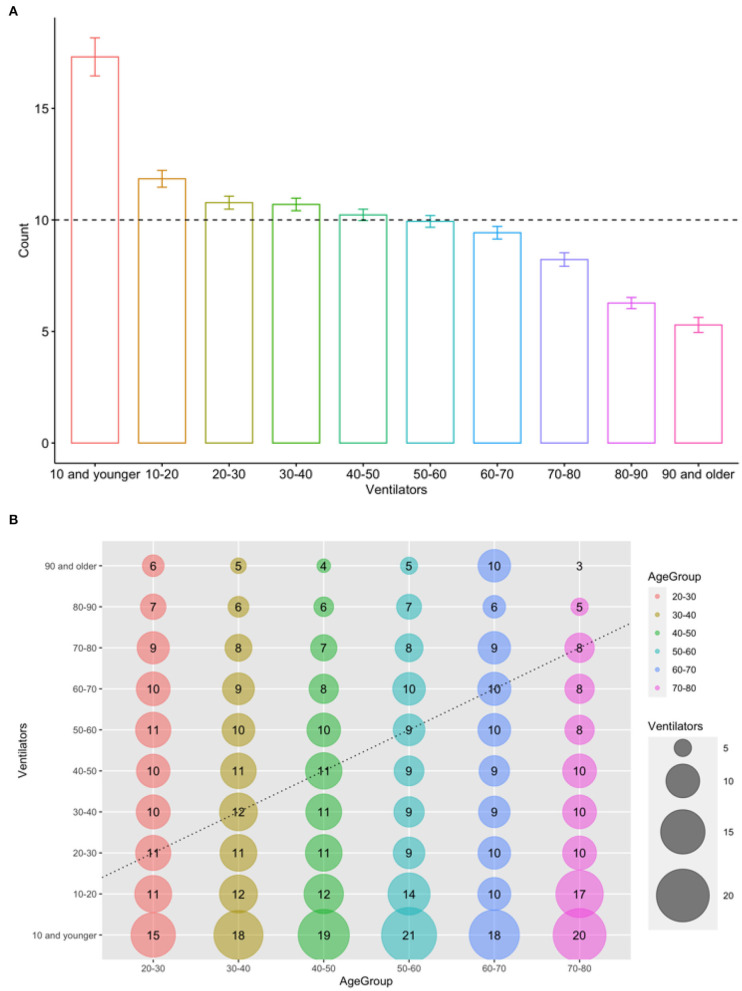
Allocations of ventilators across age groups. **(A)** The average number of ventilator allocations across patient age groups. **(B)** The average number of allocated ventilators. The x-axis represents the age groups of decision-makers (i.e., respondents), and the y-axis shows patient age groups.

Figure 1A shows the average number of ventilators allocated across age groups. Notice that the principle of *Treating patients equally* requires the allocation of exactly 10 ventilators to each age group since in the presented scenario, all patients have similar levels of severity of detectable symptoms. The other three principles would require allocating more ventilators to younger patients conditional on the assumption that younger patients have more post-treatment life-years. The results of the survey indicate that our respondents allocate more ventilators to the “0–10,” “10–20,” “20–30,” and “30–40” age groups and less ventilators for patients 60 years old or older. This finding suggests that the general public favors allocating more ventilators to younger patients, which is in conformity with the clinical ethical procedures suggested by the majority of the medical literature [see (1) for details]. Moreover, this result shows that the general public supports the ethical values adopted by some practitioners operating beyond capacity during COVID-19 (18).

Figure 1B shows that, on average, participants from all age groups allocate more ventilators to younger patients, especially to patients younger than 10, while allocating around 10 ventilators to their “own age group.” This result shows that while respondents adhere to egalitarian principles when treating their own age group (i.e., allocating around 10 ventilators), they tend to show a favoritism for the youngest age group (i.e., 10 or younger). It is noteworthy that even patients 60 or older, who receive the lowest allocation of ventilators, also favor participants who are 20 years old or younger. Table A2 shows that most socio-demographic factors and current psychological mood measures are not strong predictors of preferences over the utilization of scarce medical resources. Females demonstrate a stronger preference for allocating ventilators to younger patients, while pro-democratic participants favor younger patients with a relatively lower magnitude (19). The underlying principles followed in the construction of the allocation index by age are discussed in the Appendix.

## 4. Conclusion

COVID-19 has increased the demand for public health resources to levels unprecedented since World War II (20). Across several countries, healthcare workers had to apply strict rationing and ethical principles to efficiently utilize limited medical resources. Although the existing medical literature predominantly favors ethical rules that prioritize younger patients in terms of receiving access to scarce medical resources, the number of studies documenting the general public's views on daily clinical procedures is scant. Emanuel et al. (1) urge for the added perspective of other affected parties in the determination of existing ethical values. Our study speaks to this literature, and documents that, indeed, the general public predominantly favors younger patients, when it comes to allocation of limited number of ventilators among COVID-19 patients with similar severity of observed symptoms. We find that this result is robust to the age of the decision-makers and some other socio-demographic variables. An important limitation of our study is that we do not explicitly model the role of prognosis in medical resource allocations in our analysis. Future studies should also focus on the impact of prognosis estimations on triage decisions.

The mentioned four basic ethical principles have a binary nature and it is very likely that they may demonstrate contradictory points during practical applications. Some studies elaborate decision trees or scoring rules based on principal ethical principles that enable practitioners to use more comprehensive and unified empirical tools to maximize benefit for the greatest number of patients (21, 22). Prospective studies can develop a more comprehensive operationalization of the fundamental principles via simple decision-aiding methods. While in reality the ethical question is more complex, since patients do not always present the same severity in symptoms, our results provide useful information that aligns the general public views with the ethical standards set by the medical profession governing principles.

## Data Availability Statement

The raw data supporting the conclusions of this article will be made available by the authors, without undue reservation.

## Ethics Statement

The studies involving human participants were reviewed and approved by Texas A&M University IRB2020-0400M. The patients/participants provided their written informed consent to participate in this study.

## Author Contributions

SH, MP, and RN: study design, running the study, analyzing the data, writing the first draft, and writing the final draft.

## Conflict of Interest

The authors declare that the research was conducted in the absence of any commercial or financial relationships that could be construed as a potential conflict of interest.

## Footnotes

^1^https://www.un.org/development/desa/dpad/publication/un-desa-policy-brief-66-covid-19-and-the-least-developed-countries/

^2^https://www.cdc.gov/flu/pandemic-resources/national-strategy/planning-guidance/index.html

^3^This study was approved by Texas A&M University IRB2020-0400M and based on the IRB approved protocol requirements, personnel identifiers are removed from individual response data. Therefore, only aggregate results are reported.
