# Supplementary material for: General Public Preferences for Allocating Scarce Medical Resources During COVID-19
Source: Front Public Health. 2020 Dec 11;8:587423. doi: 10.3389/fpubh.2020.587423 (PMC7759519; doi:10.3389/fpubh.2020.587423)
Supplement: Supplementary file 1 [file Table_1.pdf]

# Appendix

Table A1: The main socio-demographic variables

| | N | Min | Max | Mean | Std. Dev. |
|---|---|---|---|---|---|
| Female | 586 | 0 | 1 | 0.40 | 0.49 |
| Age | 586 | 18 | 76 | 36.68 | 10.77 |
| White | 586 | 0 | 1 | 0.80 | 0.40 |
| Religiosity | 586 | 1 | 10 | 3.12 | 3.79 |
| Has College Degree | 586 | 0 | 1 | 0.47 | 0.50 |
| Married | 586 | 0 | 1 | 0.41 | 0.50 |
| Age of Oldest Adult | 586 | 18 | 94 | 45.27 | 15.10 |
| Approves Rep. Party | 586 | 0 | 100 | 35.38 | 35.88 |
| Approves Dem. Party | 586 | 0 | 100 | 52.86 | 33.55 |

Notes about variables: 1) Female - binary measure (1-"Yes"), 2) Age - age of participant, 3) White - binary measure (1-"Yes"), 4) Religiosity - increasing scale [0,10], 5)Has college degree - binary measure (1-"Yes"), 6) Married - binary measure (1-"Yes"), 7) Age of oldest adult - age of the oldest adult in household, 8) Approves Rep. Party - scale [0,100], 9) Approves Dem. Party - scale [0,100]

Table A2: The role of socio-demographic variables in ventilator allocations (OLS regression)

| | *Dependent variable:* | | |
|---|---|---|---|
| | index | | |
| | (1) | (2) | (3) |
| Female | 2.943* | 2.983* | 2.700* |
| | (1.553) | (1.561) | (1.582) |
| Age | 0.039 | 0.035 | 0.030 |
| | (0.079) | (0.079) | (0.079) |
| White | 5.582*** | 5.817*** | 5.874*** |
| | (1.905) | (1.906) | (1.918) |
| Religiosity | −0.132 | 0.043 | 0.106 |
| | (0.207) | (0.229) | (0.237) |
| Has College degree | 0.262 | 0.423 | 0.499 |
| | (1.507) | (1.504) | (1.509) |
| Married | 0.624 | 1.235 | 1.191 |
| | (1.644) | (1.658) | (1.664) |
| Age of Oldest Adult | 0.004 | 0.00004 | −0.006 |
| | (0.055) | (0.055) | (0.055) |
| Approves Rep. Party | | −0.059** | −0.044 |
| | | (0.027) | (0.028) |
| Approves Dem. Party | | −0.048* | −0.044* |
| | | (0.025) | (0.026) |
| Ingroup preferences | | | −0.736 |
| | | | (0.673) |
| Considers Harm consequences in decisions | | | 0.452 |
| | | | (1.021) |
| Fairness | | | 0.725 |
| | | | (1.016) |
| Constant | 52.023*** | 55.913*** | 52.621*** |
| | (3.503) | (3.914) | (5.157) |
| Observations | 586 | 586 | 586 |
| $R^2$ | 0.026 | 0.036 | 0.040 |
| Adjusted $R^2$ | 0.014 | 0.021 | 0.020 |
| Residual Std. Error | 18.037 (df = 578) | 17.977 (df = 576) | 17.984 (df = 573) |
| F Statistic | 2.212** (df = 7; 578) | 2.386** (df = 9; 576) | 1.999** (df = 12; 573) |
| *Note:* | | | *p<0.1; **p<0.05; ***p<0.01 |

Notes about variables: Since Figure 1A shows that subjects allocate 10 ventilators for middle age groups “40-50” and “50-60”) and deviate from the “Treating patients equally” principle for younger and older patients, we constructed an index to capture the changes in allocations on the tails of the distribution. Index=(“0-10”/10) *5+(“10-20”/10) *4+(“20-30”/10) *3+(“30-40”/10) *2+(“40-50”/10) *1+ (“50-60”/10) *(-1)+(“60-70”/10) *(-2)+ (“70-80”/10) *(-3) + (“80-90”/10) * (-4)+ (“90 and older”/10) *(-5)+50. The value of the index varies in the range of [0,100]. The index is sensitive to disproportional allocation on the tails. It receives a value above 50 if a respondent disproportionally favors younger patients. Conversely, if the respondent prefers older patients, then the index receives values in the range of [0,49]. A value of “0” represents full allocation to the oldest group, while a 100 represents full allocation to the youngest age group. “Ingroup preferences”, “Considers harm in decisions”, and “Fairness preferences” were constructed based on Moral Foundation Questionnaire questions (MFQ-20) [22].

Imagine that Hospital Center X has 100 available ventilators to treat COVID-19 patients. There are a total of 1000 patients seeking treatment. All of the patients are experiencing similar level of severity in their observed symptoms. The table below shows a description and the number of people seeking treatment. Your task is to assign patients to the 100 available ventilators. The total number of patients allocated cannot exceed the 100 available ventilators.

**The results of this task will be shared with Government officials. So please make your choices very carefully.**

| | |
|---|---|
| 100 patients under 10 years old. The number of ventilators I allocate for this group is: | 0 |
| 100 patients between 10-19 years old. The number of ventilators I allocate for this group is: | 0 |
| 100 patients between 20-29 years old. The number of ventilators I allocate for this group is: | 0 |
| 100 patients between 30-39 years old. The number of ventilators I allocate for this group is: | 0 |
| 100 patients between 40-49 years old. The number of ventilators I allocate for this group is: | 0 |
| 100 patients between 50-59 years old. The number of ventilators I allocate for this group is: | 0 |
| 100 patients between 60-69 years old. The number of ventilators I allocate for this group is: | 0 |
| 100 patients between 70-79 years old. The number of ventilators I allocate for this group is: | 0 |
| 100 patients between 80-89 years old. The number of ventilators I allocate for this group is: | 0 |
| 100 patients who are 90 or older. The number of ventilators I allocate for this group is: | 0 |
| Total | 0 |

**Figure A1:** Respondents were presented a hypothetical scenario, in which they had to allocate ventilators among COVID-19 patients. Only the 10% of patients had a chance to get a ventilator. The respondents were required to allocate ventilators based on 10 age groups.
